# Privacy preserving linkage using multiple match-keys

**DOI:** 10.23889/ijpds.v4i1.1094

**Published:** 2019-05-23

**Authors:** SM Randall, AP Brown, AM Ferrante, JH Boyd

**Affiliations:** 1 Curtin University, Western Australia, Perth, Australia

## Abstract

**Introduction:**

Available and practical methods for privacy preserving linkage have shortcomings: methods utilising anonymous linkage codes provide limited accuracy while methods based on Bloom filters have proven vulnerable to frequency-based attacks.

**Objectives:**

In this paper, we present and evaluate a novel protocol that aims to meld both the accuracy of the Bloom filter method with the privacy achievable through the anonymous linkage code methodology.

**Methods:**

The protocol involves creating multiple match-keys for each record, with the composition of each match-key depending on attributes of the underlying datasets being compared. The protocol was evaluated through de-duplication of four administrative datasets and two synthetic datasets; the ‘answers’ outlining which records belonged to the same individual were known for each dataset. The results were compared against results achieved with un-encoded linkage and other privacy preserving techniques on the same datasets.

**Results:**

The multiple match-key protocol presented here achieved high quality across all datasets, performing better than record-level Bloom filters and the SLK, but worse than field-level Bloom filters.

**Conclusion:**

The presented method provides high linkage quality while avoiding the frequency based attacks that have been demonstrated against the Bloom filter approach. The method appears promising for real world use.

## Introduction

Privacy preserving record linkage (PPRL) protocols involve determining which records from data collections describe the same individual where these records are encrypted or encoded so as to protect privacy. The challenge for these protocols is to allow for variations in data arising from missing, changed or incorrect identifiers (vital for ensuring a high level of matching accuracy) while at the same time ensuring that no information about the individuals within the dataset is revealed.

PPRL techniques typically adopt a semi-honest (also known as an *honest-but-curious*) model of security [1]. It is assumed that individual parties in the protocol will encode data as instructed and will not collude to leak information. However, parties can record and infer any available information, perform statistical frequency attacks on the data, use brute force attack techniques (such as dictionary attacks) to guess possible encoded values, or utilise other publicly available data to discover information about the encoded datasets [2].

**Figure 1: Privacy preserving linkage using an independent third party:**
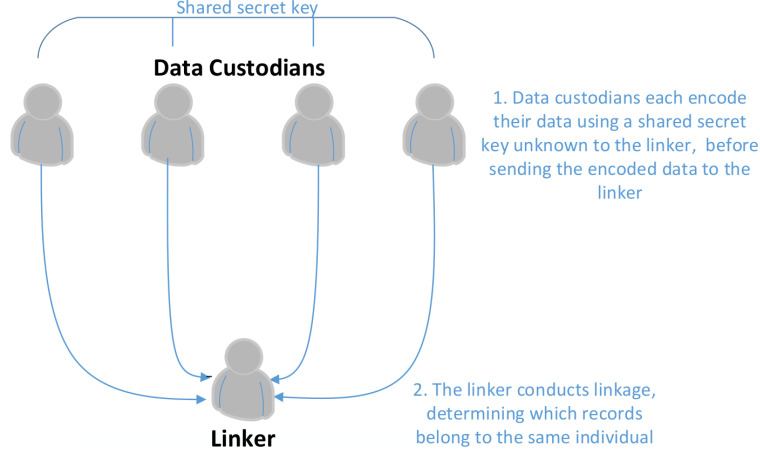


A range of techniques for PPRL have been proposed, utilizing different methodologies, and providing different levels of privacy [1]. An important distinction lies between those protocols which utilise a party independent of the data owners (three party protocols) to conduct the linkage and those which rely solely on communications between data owners for linkage to occur (two party protocols) [1]. In protocols which utilise an independent third party, data is first encoded by the data custodians before being passed to the linkage unit, who determine which records belong to the same individual (see Figure 1). This differs from two-party protocols, where data is transferred repeatedly between the two parties (the situation is more complex when involving more than two parties). Two-party protocols are significantly more complex and require greater expertise from data custodians.

Of the proposed protocols which utilise an independent third party, two approaches are prominent in the literature, each having several variants. The first involves combining particular subsets of identifiers into a hashed key which is then used in matching (referred to as a match-key [3], linkage key [4], an anonymous linkage code [5] or the minimum linkage information approach [6]). A second approach uses a structure known as a Bloom filter to store encoded information, which allows string similarity techniques to be used across encoded data.

### Anonymous linkage codes

The anonymous linkage code approach involves conducting an exact match on a pre-processed subset of personal identifiers. These identifiers are concatenated and encoded into a ‘key’ by which to identify an individual. Importantly, these methods use only a subset of identifiers. By creating a key using all available identifiers, any variation in records belonging to the same person (such as typographical errors) would result in those records being identified as belonging to different individuals. However, using too few identifiers can have the opposite effect, namely that separate individuals would be identified as the same person. This approach tries to use the optimum level of identifying information, allowing some error tolerance while correctly distinguishing between individuals [6].

Cryptographic hash functions are used to convert the concatenated identifiers into a fixed length encoded form. These hash functions have several important properties that make them suitable for this purpose. They are deterministic, meaning the same input will produce the same encoded output. They have the property that a small change in the data input will change the hash value extensively so that the new hash value does not appear correlated with the old hash value. They are also one-way functions, meaning that it is not feasible to determine the original input data when given only the hash value, other than by hashing guesses of the possible input and checking these against the original hash value [6]. To ensure adequate security, it is important that the hash function is used in combination with a secret cryptographic key which is sufficiently hard to guess [2]. The construction recommended for this purpose is known as a keyed-hash message authentication code, or HMAC (in this paper we generally use the term hash as shorthand for HMAC). This construction provides a secure way to combine a hash function with a secret cryptographic key [7]. This key should be shared amongst all data custodians and kept hidden from the linkage unit (see Figure 1). The use of a secret key prevents brute force attack techniques where an individual can guess values of concatenated identifiers, hash them, and check to see if they exist within the dataset.

There are several variants of the anonymous linkage code approach. In Australia, the Statistical Linkage Key-581 (SLK) [8] involves concatenating the second and third letters of an individual’s first name, the second third and fifth letters of their surname, and their full date of birth and sex, into a single field. This method is regularly used to link a number of national datasets. However, in practice the SLK is typically not hashed, greatly reducing its privacy protection. The Swiss Anonymous Linkage Code involves creating a hash from phonetically encoded first and last names, along with full date of birth and sex [9]. Another variant proposed by Weber [10] concatenates and then hashes the first two letters of first and last names with date of birth and sex. A method proposed by the Office for National Statistics UK (ONS) [3] extends the anonymous linkage code through the use of multiple match-keys, each made up of different combinations of personal identifiers; a match on any match-key identifies two records as belonging to the same individual.

Hashed anonymous linkage keys combined with a secret key can provide strong privacy protection. Their weakness lies in ensuring a high level of linkage quality [6]. Single linkage keys cannot tolerate differences in the identifiers selected for matching, nor can they handle missing identifiers or utilise additional available information. A number of studies have documented the lower linkage quality found through this method [11, 12], in particular the reduced sensitivity of these methods. Procedures to improve sensitivity when using the SLK have been used in practical applications; these include the use of additional variables such as address information and the splitting of the SLK back into its component fields to carry out more fine-grained matching [13]. Such procedures reduce or remove the privacy protections provided by the method.

### Privacy preserving linkage using Bloom filters

This approach involves adding encoded personal identifiers into structures known as Bloom filters (a binary array); these Bloom filters are then compared. The encoding process uses a series of hash functions (again using the HMAC construction with a secret key) to map elements of the data field to positions within the Bloom filter. The encoding process allows string similarity metrics to be used, so that variations in spelling or typographical errors can be accommodated [14].

There are two main variants of privacy preserving linkage using Bloom filters. The first is field based Bloom filters where every identifier (first name, surname etc.) is encoded into its own Bloom filter. This allows the use of techniques typical of un-encoded record linkage, such as the use of field specific weights, and the ability to appropriately handle missing values, along with previously mentioned string similarity measures. Very high linkage quality has been achieved using this method [15]. The second variant utilises a record-level Bloom filter [16] where all fields are added to a single Bloom filter which is then compared using string similarity measures. This method does not satisfactorily handle missing values [17] and uses a less sophisticated weighting approach. As such, it is likely this method yields poorer linkage quality than the field based approach, although comprehensive testing has yet to appear in print.

While Bloom filter methods have a greater tolerance for differences between records as compared to anonymous linkage codes, they also have weaker privacy protection. Both the field and record-level Bloom filter approaches have been shown to be vulnerable to frequency attacks [18-21]. While potential solutions to these attacks have been proposed [19, 22, 23], the nature of the Bloom filter method makes it difficult to ensure that further attacks will not be found. Frequency based patterns must exist within Bloom filters; these patterns are what enables the approximate matching techniques used by these methods. It is these frequency based patterns that also make it vulnerable to attacks. While solutions to these attacks remove or hide some of these frequency patterns, such patterns must always exist, and as such, it is difficult to provide any surety regarding the existence of further attacks.

### Other methods: hashing individual identifiers

Some of the earliest examples of privacy preserving record linkage involve encoding individual identifiers separately using hash functions. These encoded identifiers are then sent to the linkage unit who perform the linkage. Linkage can be carried out using standard deterministic or probabilistic methods; however, the encoding process does not allow string similarity comparisons to occur. To account for possible misspellings, techniques such as phonetic encoding can be used prior to the encoding process [24, 25].

This method of linkage has been used in Germany and France for the linkage of cancer registries. The method appears to provide high linkage quality [24, 25]; however, its weakness lies in its vulnerability to frequency attacks. For instance, it is trivial to determine the most common hash value for the ‘surname’ field, which will correspond to the most common surname in the population. As such, the privacy protections provided by these techniques are minimal.

### An alternate approach: privacy preserving linkage using multiple match-keys

In this paper we present an alternate methodology to the approaches described above. The proposed method seeks to combine the best features of both approaches: namely, the privacy protection offered by the use of anonymous linkage code along with the linkage quality offered by the Bloom filter approach. An approach that could achieve linkage quality similar to the Bloom filter method without the associated privacy risks would be highly desirable [26].

Unlike the Bloom filter approach, our method does not make use of approximate string matching. Rather it aims to achieve high quality linkage through utilising other important techniques from traditional (un-encoded) probabilistic linkage including the use of weights and methods for managing missing values.

## Methods

### Overview of the protocol

#### Overview

The proposed method extends the anonymous linkage code approach. For each record, a number of hashes are created, each from different sets of concatenated personal identifiers; we refer to these hashes as ‘match-keys’. Any pair of records with the same value for any particular match-key are identified as belonging to the same individual; that is, each match-key will directly identify an individual. Rather than use a predetermined set of match-keys, match-keys are generated based on parameters which describe the underlying characteristics of the data. These parameters are shared between the data custodians, and are used as input to the encoding process. Once encoded, data is sent to the linkage unit for linkage.

The number of match-keys and the composition of each match-key are all determined as part of this privacy preserving approach. Importantly, this composition differs depending on the characteristics of the dataset(s) in question. These parameters are identified through utilizing methods from probabilistic record linkage.

#### Probabilistic linkage methods

Probabilistic linkage uses conditional probabilities to compute the likelihood of two records belonging to the same person [27]. Records are compared on a pairwise basis. A comparison of two records involves comparing each field. Each field comparison results in a score based on whether the fields do or do not match (known as the agreement and disagreement weight, respectively). The field scores are then summed; if the summed score exceeds a specific threshold, the two records are deemed a match [27]. Field scores are calculated using two conditional probabilities, known as *m* and *u* probabilities. The *m-probability* is the likelihood that two records belonging to the same person have the same value for a particular field. The *u-probability* is the likelihood that two records belonging to different people have the same value for a particular field [27]. These are converted into agreement and disagreement weights using the following formulas:

$$\text{Agreement Weight}=\log\left(\frac{m}{u}\right)$$

$$\text{Disagreement Weight}=\log\left(\frac{1-m}{1-u}\right)$$

Numerous techniques exist for estimating *m* and *u* probabilities for a particular dataset and for estimating the designated threshold [28]. These include Jaro’s method for estimating u-probabilities, the expectation-maximisation estimation algorithm [29] and the iterative refinement procedure first described by Newcombe [30].

#### Methodology

From the basic probabilistic model, it is possible to iterate through all possible combination of field state comparisons for a pair of records [28]. We will consider a simplified model, whereby a field comparison can either agree or disagree. The total number of different combinations of field state comparisons is then two to the power of the number of fields (i.e. the total number of field state comparisons doubles with the introduction of another field).

Using estimated *m* and *u* probabilities and an estimated threshold score, we can calculate the exact total score each combination of field comparisons would receive, and determine which would score above the threshold (an example is shown in Table 1) [28].

**Table 1: A list of field state combinations (16 different states are possible as there are four fields):** 

	First Name	Surname	Sex	Year of Birth	Summed Score

1	Agree	Agree	Agree	Agree	17
2	Agree	Agree	Disagree	Agree	15.5
3	Disagree	Agree	Disagree	Agree	10
	…	…	…	…	…

The proposed method replicates the combinations that occur above the threshold in a privacy preserved manner. The encoding process is simple; for each field state comparison, a match-key is created from hashing a concatenation of each field comparison in agreement. These hashes use the HMAC construction with a secret key shared between data providers, as in Figure 1. If one of the component fields to be concatenated is missing, the match-key is left blank. An example of this process, using example data and the combinations from Table 1, is shown in Table 2. Any two records with the same value for a particular match-key are designated a match.

**Table 2: An example of the encoding process: two un-encoded records (top) are encoded (bottom) using the field state combinations from Table 1:** 

Original Data				

Record ID	First Name	Surname	Sex	Year of Birth

Record1	Sean	Randall	M	1986
Record2	John	Doe		1957

Encoded Data			

Record ID	Match-Key1	Match-Key2	Match-Key3

Record1	HMAC(SeanRandallM1986)	HMAC(SeanRandall1986)	HMAC(Randall1986)
Record2		HMAC(JohnDoe1957)	HMAC(Doe1957)

#### Reducing the number of match-keys

The method as described creates match-keys for every field combination above the threshold. This can result in a large number of match-keys per record, creating large encoded datasets and increasing computational load. However, a great number of these created match-keys are redundant. For instance, if a match-key made up of encoded first name, surname and date of birth is considered identifying, then there is no need to also compute a match-key for first name, surname, date of birth and sex; no additional matches could possibly be found. In this way we can remove a large number of field combinations without affecting results. To identify the redundant field combinations is straightforward; given a field combination with a set of *x* fields marked as ‘Agree’ (see Table 1), any other field combinations that also contains that same set of *x* fields marked as ‘Agree’ are not required. This procedure can be applied iteratively over all field combinations to remove all redundant field comparisons (example code to encode match-keys from raw data is provided as supplementary material).

Preliminary testing suggests this method can greatly reduce the number of match-keys required. A typical example of a linkage involving nine fields produced 402 field state combinations over the given threshold; after removal of unnecessary combinations, only 41 match-keys were required.

In the next sections, we evaluate this simple method on a range of synthetic and real administrative datasets. We compare the results against those achieved with un-encoded linkage and against other privacy preserving techniques.

### Evaluation methodology

#### Evaluation strategy

De-duplication linkages were undertaken on a range of synthetic and real administrative datasets. Each dataset had either a truth-set available (for the synthetic datasets) or a gold standard benchmark with which to compare results (for real datasets). A range of different linkage methods were compared, including both un-encoded and privacy preserving methods. The un-encoded methods included probabilistic record linkage using approximate string matching and probabilistic linkage using exact matching only. Privacy preserving methods tested comprised field-level Bloom filters, record-level Bloom filters, the SLK-581 and the multiple match-key methodology. Parameters for each linkage method were calculated using the available truth-sets and gold standard benchmarks, with results reported at the threshold providing the optimal linkage quality (where F-measure was maximised). Parameters were shared across methodologies where possible. Results were compared using the precision and recall measures described below. Algorithms were implemented in Python 2.7 [31]; linkage was conducted using the LinXmart linkage engine [32], which implements the standard probabilistic linkage methodology.

#### Datasets

Six separate datasets were included in the evaluation; two synthetic datasets and four real-world administrative datasets.

The two synthetic datasets ‘FER12’ and ‘BRO17’ have been used in previously published research, and detailed information on the data generation process is available [28, 33]. The FER12 dataset contained 400,000 records, of which an individual could at most have 6 duplicate records; fields included first and last name, date of birth, sex, and postcode. Each field had its own rate of errors and distribution of types of errors. The BRO17 dataset contained 1,000,000 records; the distribution of records per person was modelled on a hospital morbidity data collection with a ‘long tail’ where a small number of individuals had hundreds of records per person. The BRO17 dataset had exactly 10% of fields randomly set to missing, and another 10% of fields modified in some way (by truncation, misspellings, replacement of values, etc). Fields included first name, middle name, last name, date of birth, street address, and postcode. Each of these datasets contained the ‘answers’, identifying which records did belong to the same individual. Both datasets are available from the authors on request.

Four large-scale Australian health datasets were also used in this evaluation; these were hospital admission records from New South Wales (NSW) and South Australia (SA), and emergency department presentations from NSW and SA. Each dataset contained all records from the three years 2008-2010; only public hospital data was available in the South Australian datasets. Each dataset had previously been de-duplicated to a high quality by jurisdictional linkage units (the Centre for Health Record Linkage and SANT Data Link for NSW and SA datasets respectively); the links created by these units were used as the gold-standard benchmark against which our de-duplication results were compared. These linkage units utilised a variety of deduplication methods including intensive manual review of created links along with quality assurance procedures to analyse and review potential errors [34]. The links created by these linkage units have been further validated through their regular use in academic and government research. The data (personal identifiers only) was made available as part of a Proof of Concept project for the Population Health Research Network [35]; ethics approvals were obtained from SA Health, the Cancer Institute NSW and Curtin University.

Each dataset contained name information (first name, middle name and surname), sex, date of birth, and address information (street address and postcode). Fields used for linkage and the percentage of missing values within each dataset are described in Table 3.

**Table 3: Number of records and percentages of missing values for each dataset:** 

	FER12	BRO17	SA Emergency	NSW Emergency	SA Hospital	NSW Hospital

No. Records	400,000	1,000,000	813,839	4,304,459	1,007,242	6,658,380
*Proportion of missing values*						
First Name	2.4%	10.0%	2.2%	0.1%	3.1%	33.2%
Middle Name	-	10.0%	74.4%	83.4%	79.3%	66.9%
Surname	2.6%	10.0%	1.3%	0.0%	2.4%	33.3%
Date of Birth	11.8%	10.0%	0.0%	0.0%	0.0%	0.0%
Sex	5.2%	10.0%	0.0%	0.0%	0.0%	0.0%
Address	-	10.0%	4.6%	4.2%	7.8%	10.4%
Postcode	1.1%	10.0%	7.5%	1.2%	9.4%	0.6%

#### Linkage methods

Each dataset was de-duplicated using a range of linkage techniques; no linkages were conducted between any of the datasets. The same weights and blocking methods were used across linkage techniques, and multiple threshold scores were tested for each method (not all techniques required blocking, weights or thresholds). Agreement and disagreement weights were calculated directly from the available gold standard benchmark/truth-set. Two sets of blocks were used; Soundex of surname concatenated with sex, and full date of birth. This linkage strategy was based on a previously published ‘default’ strategy that has been regularly used in linkage evaluations [36, 37].

Probabilistic linkage was carried out using un-encoded identifiers. All available variables were used in comparisons. Two probabilistic linkages were carried out; the first used the Jaro-Winkler string similarity metric [38] for alphabetic variables (names and address) and exact matching for other variables; the second used exact matching for all variables.

Field based Bloom filters were created according to a previously published methodology [14, 15]. Bloom filters were 100 bits in length, with each variable split into bigrams that were hashed and added to the Bloom filter; three hashes were created for each bigram. The Sorenson-Dice coefficient [39] was used to compare Bloom filters. Weights and blocking fields were used as described above.

Record based Bloom filters were created based on the cryptographic long-term key construction by Schnell [16], using the weighting method described by Durham [40]. For each record, a Bloom filter of 1000 bits was created. The number of hashes computed for each bigram in each field depended on the weight of the field, as well as the average length of the field. Address information was not added to record-level Bloom filters as preliminary testing indicated reduced linkage quality when these fields were included; previous research has also noted this issue [17]. The middle name field was also excluded due to its high proportion of missing values. Separate blocking fields were also created as described above.

The standard SLK-581 was also evaluated, created from the second and third letters of the individual’s first name, the second third and fifth letters of their surname, along with full date of birth and sex [4].

For the multiple match-key algorithm, weights were used to generate field state combinations. Linkage quality was calculated on all generated match-keys over the chosen threshold. The SHA-1 hash algorithm was used with output truncated to 90 bits per hash; this provided adequate security against collisions (for 100 million unique hash values there was approximately a 1 in a trillion chance of two hashes having the same value) while reducing file sizes.

#### Measuring linkage quality

Linkage quality was measured using pairwise precision and recall, with the F-measure used as an overall metric of linkage quality. Results were reported at the threshold which maximised the F-measure.

## Results

Linkage quality results for each tested linkage method across all six datasets are shown in Table 4; results are shown at the threshold which optimised linkage quality.

**Table 4: Results from linkage quality evaluation:** ^1^ Privacy preserving record linkage ^2^ Statistical linkage key

Dataset 1: FER09		Precision	Recall	F-measure

New PPRL1 method	Multiple match-key PPRL	0.928	0.788	0.856
PPRL	SLK2	0.871	0.570	0.689
PPRL	Record-level bloom filter	0.937	0.778	0.850
PPRL	Field-level bloom filter	0.941	0.793	0.860
Un-encoded	Probabilistic linkage using approximate string matching	0.986	0.805	0.886
Un-encoded	Probabilistic linkage using exact matching only	0.940	0.777	0.851

Dataset 2: BRO17		Precision	Recall	F-measure

New PPRL method	Multiple match-key PPRL	0.992	0.943	0.967
PPRL	SLK	0.960	0.239	0.383
PPRL	Record-level bloom filter	0.934	0.691	0.794
PPRL	Field-level bloom filter	0.997	0.813	0.896
Un-encoded	Probabilistic linkage using approximate string matching	0.996	0.815	0.897
Un-encoded	Probabilistic linkage using exact matching only	0.993	0.810	0.892

Dataset 3: SA Emergency		Precision	Recall	F-measure

New PPRL method	Multiple match-key PPRL	0.967	0.990	0.978
PPRL	SLK	0.995	0.945	0.969
PPRL	Record-level bloom filter	0.992	0.956	0.974
PPRL	Field-level bloom filter	0.984	0.978	0.981
Un-encoded	Probabilistic linkage using approximate string matching	0.985	0.980	0.982
Un-encoded	Probabilistic linkage using exact matching only	0.969	0.990	0.979

Dataset 4: NSW Emergency		Precision	Recall	F-measure

New PPRL method	Multiple match-key PPRL	0.997	0.983	0.990
PPRL	SLK	0.999	0.966	0.982
PPRL	Record-level bloom filter	0.989	0.978	0.983
PPRL	Field-level bloom filter	0.995	0.987	0.991
Un-encoded	Probabilistic linkage using approximate string matching	0.995	0.990	0.993
Un-encoded	Probabilistic linkage using exact matching only	0.995	0.985	0.990

Dataset 5: SA Hospital		Precision	Recall	F-measure

New PPRL method	Multiple match-key PPRL	0.993	0.991	0.992
PPRL	SLK	0.975	0.988	0.981
PPRL	Record-level bloom filter	0.991	0.992	0.992
PPRL	Field-level bloom filter	0.995	0.989	0.992
Un-encoded	Probabilistic linkage using approximate string matching	0.996	0.987	0.992
Un-encoded	Probabilistic linkage using exact matching only	0.995	0.988	0.991

Dataset 6: NSW Hospital		Precision	Recall	F-measure

New PPRL method	Multiple match-key PPRL	0.983	0.991	0.987
PPRL	SLK	0.072	0.920	0.134
PPRL	Record-level bloom filter	0.754	0.921	0.829
PPRL	Field-level bloom filter	0.992	0.989	0.990
Un-encoded	Probabilistic linkage using approximate string matching	0.992	0.989	0.991
Un-encoded	Probabilistic linkage using exact matching only	0.988	0.991	0.990

As expected, un-encoded probabilistic record linkage using approximate string matching achieved the highest linkage quality across all datasets. Generally, the use of approximate string matching as compared to exact matching resulted in minor decreases in linkage quality; this decrease was larger for the synthetic datasets, likely due to their higher rates of error.

In regards to privacy preserving techniques, field-level Bloom filters provided the highest linkage quality on all but one of the tested datasets. The multiple match-key method was the next best in terms of quality, with results only slightly below those for the field-level Bloom filters on most datasets. The record-level Bloom filters typically performed below that of the multiple match-key method, except for the SA Hospital dataset, where all three of these PPRL methods performed equally. The SLK method performed adequately on three of the four administrative datasets, however, results were lower than for all other tested methods. This method performed notably poorer for the NSW hospital dataset and both synthetic datasets due to the preponderance of missing values in these files.

One notable outlier was the results from the BRO17 dataset, where the multiple match-key method outperformed all compared methods, including un-encoded methods. We attributed this to the fact that the multiple match-key method does not require blocking; the BRO17 dataset had high levels of missing values in all fields and the standard blocking strategy was likely not appropriate here.

The number of hashes created in the multiple match-key method for each dataset varied from 14 (FER12 synthetic data) to 83 (NSW hospital data). Time taken for data encoding and linkage, and encoded file sizes (not reported but available from authors) were comparable to other evaluated methods.

## Discussion

In general, the privacy preserving linkage methods evaluated here showed high linkage quality, providing continuing evidence of the viability of this method of record linkage. This was particularly apparent in datasets with few missing values or errors in identifiers, where all tested methods provided very high linkage quality.

Based on these results, field-level Bloom filters are the privacy preserving method which provides the greatest linkage quality. The high quality returned from our linkages were consistent with those achieved previously [15]. However as previously mentioned this method is vulnerable to frequency attacks [18-21] and so may not be suitable in situations where privacy protection is paramount. As expected, record-level Bloom filters performed poorly when compared against their field-level equivalents, and also performed poorly relative to the multiple match-key method introduced here.

In contrast, while the SLK method is simple to implement and can provide strong privacy protection if used appropriately (i.e. using the HMAC algorithm with a strong password), it does not appear suitable as an all-purpose privacy preserving linkage method, given the very poor linkage quality seen with some of the datasets. Although not tested here, we expect other anonymous linkage code methods to perform similarly to the SLK.

The multiple match-key method introduced in this paper provided admirably high linkage quality. It was superior to the SLK method, which was the only evaluated privacy preserving method with similar privacy protections. The field level Bloom filter was the only privacy preserving method to produce higher linkage quality; this method has known deficits in terms of its privacy protections [18-21]. It was not unexpected that field-level Bloom filters provided higher quality results, given their additional use of approximate string matching to identify matches; however, the associated increase was typically small in magnitude.

A key consideration in assessing the viability of the multiple match-key privacy preserving method was determining the extent to which string similarity matching (which this method does not use) contributes to high linkage quality. Previous studies comparing results using string similarity matching to those without have found large decreases in error rates for some datasets [38]. A number of publications (including those of the authors [6]) have stressed the importance of approximate matching methods for ensuring accurate privacy preserving record linkage. However, this study has found the difference between un-encoded linkages utilising approximate matching and those using only exact matching to be small, suggesting the importance of string similarity matching in ensuring quality may be overstated. The extent to which string similarity metrics improve results will clearly depend on the characteristics of the dataset in question; in an extreme example, Winkler reports a linkage in which among true-matches 20% of last-names and 25% of first names contained spelling differences [41]. Such a dataset clearly would require approximate matching techniques, and we would expect our multiple match-key method to perform poorly here. It is an open question as to what proportion of administrative datasets fall into this category.

### Privacy of the multiple match-key PPRL method

The multiple match-key method presented here appears highly resistant to both dictionary and frequency attacks. Dictionary attacks are not possible through the use of a secret key in hashing (the HMAC construction) which is shared amongst data custodians and kept from the linkage unit. Frequency attacks also do not appear possible. Each particular match-key generated by this protocol is made up of a combination of fields that directly identifies an individual. If the same value of a match-key exists in two or more records, this means these records belong to the same person. As such any frequency analysis of match-keys will simply provide a list of which individuals who are found in the datasets most often, rather than provide any information on their identifiers.

The hash-based encoding process used in this protocol means that similar input values do not result in similar match-keys, a feature of the Bloom filter approach which has allowed frequency attacks to occur. As the protocol does not create match-keys if one of their component fields is a missing value, it is also not possible to perform frequency attacks of match-keys on the subset of records where particular fields are missing. The use of inappropriate match-keys (for instance, the use of the single surname field as a match-key) would allow frequency attacks to occur. This could potentially occur through human or other error. Such a match-key is not advisable not just on privacy grounds but also on quality grounds, as it would of course also result in extremely poor linkage quality (all records with the same surname would be matched together). In practice, this type of error would be easy to identify before data is encoded and sent to the linkage unit, and so is unlikely to occur.

The use of more than one match-key provides one vector by which information about the individuals can be learnt. Information is leaked when comparing two records with some match-keys matching and others not-matching. For instance, if two records have the same match-keys for combinations that do not include surname, but different match-keys for combinations that do include surname, it is likely that the surnames differ between these matching records. This can reveal information about the record in question; for instance, as it is more common for women than men to change surname in their lifetime, we could guess that this record is more likely to be female than male. While the use of multiple match-keys can leak information, it does not appear able to re-identify an individual; rather, it suggests broad demographic groupings of which a record may be part. This privacy issue is not unique to the multiple match-key method but is inherent in all privacy preserving methods which use multiple encoded values. In situations where greater privacy considerations are required such that no information about an individual can be inferred, a single match-key (i.e. the SLK approach but using the HMAC construction) is the most viable option, despite its associated reduction in linkage quality.

### Strengths and limitations

The privacy preserving method presented here achieves both high accuracy and appears to provide strong privacy protection. While the absence of approximate string matching in the method may present as a limitation, our results suggest that, in general, approximate string matching provides limited quality improvement. However, for certain datasets, approximate matching will be of greater importance, such as those with very few identifiers or large numbers of typographical errors, and in these situations, we expect the multiple match-key protocol will likely perform worse than other techniques such as record-level Bloom filters.

The method proposed here is an extension of the anonymous linkage code concept to utilise more than one match-key. A similar method has been proposed by the ONS [3], although it has yet to be evaluated. A key difference is that in our method, the generation of match-keys is based on underlying characteristics of the datasets while the ONS approach uses a set of predetermined match-keys for all datasets. By generating match-keys in this way, our method will be applicable to a wider range of datasets, including those containing fields with large proportions of missing values and those with additional or alternate fields to the ones specified in the hard-coded method.

Further research is needed to investigate the performance of the multiple match-key method (as well as the other methods) in a real-world setting, where parameters must be estimated rather than calculated. Techniques for estimating weights and thresholds necessary for the multiple match-key methodology exist and have received evaluation in privacy preserving contexts [28]. It should be noted that such parameters are normally estimated by the linkage unit at time of linkage; however, the proposed protocol requires estimation prior to data transfer, as estimated parameters are used in data encoding. A simple method to generate these parameters would be for each data custodian to compute parameters for their datasets and provide these to the linkage unit, who can then calculate a set of global parameters to be used for encoding all datasets, based on these local parameters. Additional work is required to validate such a procedure.

## Conclusion

In this paper we describe and evaluate a new approach to PPRL. The results of our evaluation suggest this method can achieve very high quality results, while at the same time providing strong privacy protection.

The differing privacy preserving protocols evaluated in this paper each have their own strengths and weaknesses, and will each be suitable in particular scenarios. The multiple match-key protocol does not achieve as high a quality as field-level Bloom filters but offers greater privacy protection. It provides slightly better linkage quality in most scenarios as compared with the record-level Bloom filter approach, while providing greater certainty regarding privacy. Finally, it provides greater linkage quality than that offered by a solitary match-key such as the SLK method. As such, we feel this protocol is an important and timely contribution to the current state of the art.

## Ethics

This study received ethical approval under the Population Health Research Network’s Proof of Concept project, which included approval for developing and refining linkage methodology. Approval was obtained from Curtin University Human Research Ethics Committee as well as from New South Wales Cancer Institute Human Research Ethics Committee and South Australian Department of Health and Aging Human Research Ethics Committee.

## Abbreviations

NSW	New South Wales
ONS	Office for National Statistics UK
PPRL	Privacy preserving record linkage
SA	South Australia
SLK	Statistical linkage key
